# Dr. E. Magitot

**Published:** 1897-11

**Authors:** 


### OBITUARY.

### Dr. E. Magitot.

The Odontological Society of Chicago, recognizing the great services rendered by Magitot for the advancement of dental science, has adopted and ordered sent to the dental journals of the United States and France, and to the family of the deceased, the following:

Magitot was born in Paris in 1833, and died there during the current year. His first contribution to dental literature was made in 1857 at the age of twenty-four, relating to the structure and development of the human teeth, while the last came from his pen in 1897, just before he died. During these forty years Magitot wrote no less than sixty-five books, essays, pamphlets, etc., dealing exclusively with nearly every phase of dental embryology, histology, biology, pathology, hygiene, etc. No writer of any age has made as many, as varied and as valuable contributions to dental science, as Magitot.

The priceless services rendered by him entitle him to rank as one of the foremost investigators in odontology. He was a member of numerous scientific bodies and societies, whose members sincerely mourn his loss. It may be truly said that when Magitot passed from the scenes of human activity, dental science, not of France alone, but of the entire world, lost one of its noblest and greatest minds.

The dental profession of the United States recognizing and appreciating Magitot's services, keenly mourns and sympathizes with his bereaved family and the profession of France, by reason of his demise.

A. W. HARLAN,<br>

J. W. WASSALL, } *Committee.*<br>

LOUIS OTTOFY,

CHICAGO, September 1, 1897.

